# The relationship between prices and output in the UK and the US

**DOI:** 10.1007/s43546-022-00231-4

**Published:** 2022-05-09

**Authors:** Guglielmo Maria Caporale, Gloria Claudio-Quiroga, Luis Alberiko Gil-Alana

**Affiliations:** 1 Department of Economics and Finance, Brunel University, London, UB8 3PH UK; 2 Universidad Francisco de Vitoria, Madrid, Spain; 3 University of Navarra, Pamplona, Spain

**Keywords:** Real output, Prices, Persistence, Fractional integration, C22, C23, E32

## Abstract

This paper aims to provide new evidence on the relationship between prices and output in both the US and the UK (which is important to discriminate between different macroeconomic theories) by focusing on the long run. For this purpose, it applies fractional integration and long-range dependence techniques that are more general than the standard modelling approach based on the stationary *I* (0) versus nonstationary *I* (1) dichotomy which has been used in previous studies. All series appear to be highly trended and to exhibit high degrees of integration and persistence, especially in the case of CPI. Since the two variables have different degrees of integration in each of the two countries, fractional cointegration tests cannot be carried out. We assume instead weak exogeneity of each of them in turn and test for causality by regressing the other variable against lagged values of the weakly exogenous one. We find that the only significant relationship implies the existence of a lagged effect of prices on output in the case of the US, which suggests a dominant role for demand shocks.

## Introduction

The relationship between prices and output is crucial to understanding the nature of economic fluctuations and to be able to discriminate between rival macroeconomic models. A lot of the literature in this area has focused on whether prices are procyclical or countercyclical, i.e. whether they move in the same or in the opposite direction to output. This depends on the nature of the underlying shocks: aggregate demand and/or monetary policy shocks should produce procyclical behaviour, whilst aggregate supply (technology) shocks should result in countercyclical behaviour. Friedman and Schwartz (1963, 1982) analysed US business cycles from the Civil War and concluded that monetary shocks were the main source of aggregate fluctuations and that prices were procyclical, a stylised fact that macro models had to be able to replicate to be data congruent (Bernanke 1986; Mankiw 1989). Such price behaviour is consistent with the Phillips Curve relationship and other models based on monetary surprises.

By contrast, in the Real Business Cycle (RBC) framework introduced by Kydland and Prescott (1990) business cycles are defined as deviations from trend (as in Lucas 1977), technology shocks are the main driver of cycles and the correlation between the cyclical component of output and the price level was estimated to be negative. However, Cooley and Ohanian (1991) found a positive correlation between output and inflation (as opposed to prices) during the post-war period.

On the whole, the evidence concerning the US is mixed (see, e.g., Lee 2006; Konstantakopoulou et al. 2009; Haslag and Hsu 2012; Brock and Haslag 2014; Keating and Valcarcel 2015). Some studies argue that this might depend on the different sample periods used for the analysis, as the nature of the macro shocks driving cycles might have changed (see Backus and Kehoe 1992, and Smith 1992). Lee (2006) used the DCC-GARCH model to examine the dynamic correlation between US prices and output and found that they tended to move in the same direction before World War II but in the opposite direction afterwards. Antonakakisa et al. (2017) analysed the time-varying correlation between US output and prices by incorporating short-term interest rates, output and inflation volatilities in the model to capture the role of monetary policy, output and inflation uncertainty; they found evidence of time variation and of a predominant role for technology shocks.

Concerning the international evidence, Backus and Kehoe (1992) considered ten countries with data spanning at least a century (Australia, Canada, Denmark, Germany, Italy, Japan, Norway, Sweden, UK and US) and found that prices were more persistent and generally procyclical before World War II (WWII) and countercyclical afterwards; however, in the case of the UK the correlation between the growth rates of output and growth, though positive, was relatively small in the earlier period and sensitive to the estimation method for output. Vázquez (2002) studied the co-movement between output and prices in the EU15 countries and found that the UK and others nine countries (Austria, Belgium, France, Germany, Greece, Italy, Luxembourg, Spain, and Sweden) exhibit a negative correlation between prices and output in the long run; in short-run the correlation becomes positive for France, Italy, and Portugal; the remaining four countries, namely Denmark, Finland, Ireland, and the Netherlands do not display any significant co-movement between prices and output. Smith (1992) found that prices behaved procyclically before World War I (WWI) and countercyclically after the Great Depression in the US, the UK, Canada, Australia, Sweden, Italy, Denmark, Norway, Japan, and Germany.

Den Haan and Summer (2004) analysed the correlation coefficients in the G7 at different forecast horizons using a VAR methodology as in Den Haan (2000) and estimated positive short-term and negative long-term correlations in the post-WWII period. Fiorito and Kolintzas (1994) also studied the G7 countries and concluded that the correlation between HP-filtered prices and output is negative during the post-WWII period. Finally, Pollin and Zhu (2006) analysed the relationship between inflation and economic growth in 80 countries over the 1961–2000 period; their results vary across countries but suggest a stronger positive correlation during periods characterised by more active demand management policies.

Some more recent studies find unidirectional causality. For example, Mahmoud (2015) reports that causality runs from inflation to economic growth in Mauritania, while Denbel et al. (2016) detects causality running in the opposite direction in Ethiopia. Other papers estimate a long-run equilibrium, but do not provide clear evidence regarding the direction of causality. For instance, Raghutla et al. (2019) find a negative long-run relationship between inflation and output growth in India, Kassim and Manap (2017) in Malaysia, and Gatawa et al. (2017) and Onwubuariri et al. (2021) in Nigeria: also, Ahmmed et al. (2020) detect a positive one in Malaysia, Thailand, Singapore, Japan, and Bangladesh, and a negative one in the US, Pakistan, the UK, and India; finally, Anochiwa and Maduka (2015) find a nonlinear relationship in Nigeria.

The present paper aims to provide new evidence on the relationship between prices and output in both the US and the UK by focusing on the long-run rather than on cyclical fluctuations. For this purpose, it applies fractional integration and long-range dependence techniques; these are more general than the standard modelling approach based on the stationary *I* (0) versus nonstationary *I* (1) dichotomy which has been used in previous studies since they allow the differencing parameter to be a fractional value as well as an integer and, therefore, are more general and flexible. After analysing the stochastic behaviour of each individual series, we examine their long-run linkages. Since the two variables have different degrees of integration in each of the two countries, fractional cointegration tests cannot be carried out for this purpose. We assume instead weak exogeneity of each of them in turn to carry out causality tests in a regression framework. Please note that the key contribution of the paper is represented by the chosen methodology which, as already mentioned, uses a more general modelling approach than the papers discussed above and enables us to establish both whether the effects of shocks to the individual series are transitory or permanent and whether they are linked by a long-run equilibrium relationship; instead it is not our aim to analyse instead the determinants of either inflation or output.

The rest of the paper is structured as follows: Sect. Methodology outlines the methodology used for the analysis which is based on the concepts of fractional integration. Section Data and empirical results describes the dataset and presents the empirical results. Section Conclusions offers some concluding remarks.

## Methodology

Stationarity is a crucial concept in time series econometrics. In particular, a series is said to be covariance (or second order) stationary if its first two moments are independent of time. However, most macroeconomic series appear to be nonstationary. A standard approach to remove nonstationarity is to take first differences on the assumption that the differenced series will be stationary *I* (0). In such a case the original series is said to be integrated of order 1 or *I* (1). Following the seminal work of Nelson and Plosser (1982) many papers have, therefore, carried out standard unit root tests.^1^ However, it is now well known that such tests have very low power under fractional alternatives,^2^ as it is possible for a series to be neither *I* (0) nor *I* (1) but instead integrated of order d, where d can be any fractional value in the interval between 0 and 1, or even to be above 1. Gil-Alana and Robinson (1997) examined an updated version of Nelson and Plosser’s (1982) dataset consisting of fourteen US macro variables and found that all except one were *I* (*d*) with 0 < *d* < 1. Since then, fractional integration has been widely used for the analysis of macro series (see, e.g., Mayoral 2006; Chambers 1998; Michelacci and Zaffaroni 2000; Caporale and Gil-Alana 2013; Abbritti et al. 2016).

Therefore, the model estimated in the empirical section is of the following form:

1 $$(1\,\, - \,\,L)^{d} x_{t} = u_{t} ,\,\,\,\,\,\,\,\,\,t\, = 1\,,\,\,2\,,\,\,...\,,$$

where *x*_*t*_ stands for either the observed data or the errors in a regression model that may include deterministic terms such as a constant or a linear time trend or weakly exogenous variables, *d* is a parameter to be estimated from the data providing a measure of persistence, and *u*_*t*_ is an *I* (0) process that is assumed to be in turn a white noise or exhibiting (weak) autocorrelation. Note that the specification given by (1) includes the classical cases of *I* (0) and *I* (1) if *d* = 0 and 1, respectively, and if *u*_*t*_ is an AutoRegressive Moving Average (ARMA) process with orders *p* and *q* for the AR and MA components, *x*_*t*_ becomes an ARMA (*p*, *q*) and ARIMA (*p*, *q*, *q*) process, respectively, and an ARFIMA (*p*, *d*, *q*) one for non-integer values of d. By allowing d to take any real value, we make the model sufficiently general to include a variety of cases such as those of nonstationary though mean-reverting series when d belongs to the interval [0.5, 1]. The estimation of the differencing parameter *d* will be based on the Whittle function in the frequency domain using a parametric approach developed in Robinson (1994). This methodology allows to consider any value of d including nonstationary cases (*d* ≥ 0.5) and thus it does not require preliminary differentiation in the case of nonstationary data; it has a standard *N* (0, 1) limit distribution and it is the most efficient method in the Pitman sense against local departures from the null.

Fractional cointegration is the extension of the concept of fractional integration to the multivariate case. A necessary condition to test for it in a bivariate context such as ours is that the two individual series display the same degree of integration. Since this condition is not satisfied in our case (see below) cointegration tests cannot be performed; instead we analyse the relationship between the two variables by treating each of them in turn as weakly exogenous, i.e. the other variable is regressed against lagged values of the weakly exogenous one; in other words, we consider the following model:

2 $$y_{1t} = \alpha + \beta \,y_{2t - k} + \,x_{t} \,,\,\,(1\,\, - \,\,L)^{d} x_{t} \, = u_{t} ,\,\,\,\,\,\,\,t = 1\,,\,\,2\,,\,\,...\,,$$

for *k* > 0, where *y*_1*t*_ and *y*_2*t*_ stand for log CPI and log real GDP, respectively. We carry out the analysis using a simple version of the tests of Robinson (1994) that allows the inclusion of deterministic or weakly exogenous regressors in a model where the errors are potentially *I* (*d*) and *d* may be any real value. In this context, the limit distribution is unaffected by the inclusion of these terms or the specification of the *I* (0) error term.

## Data and empirical results

**Fig. 1:**
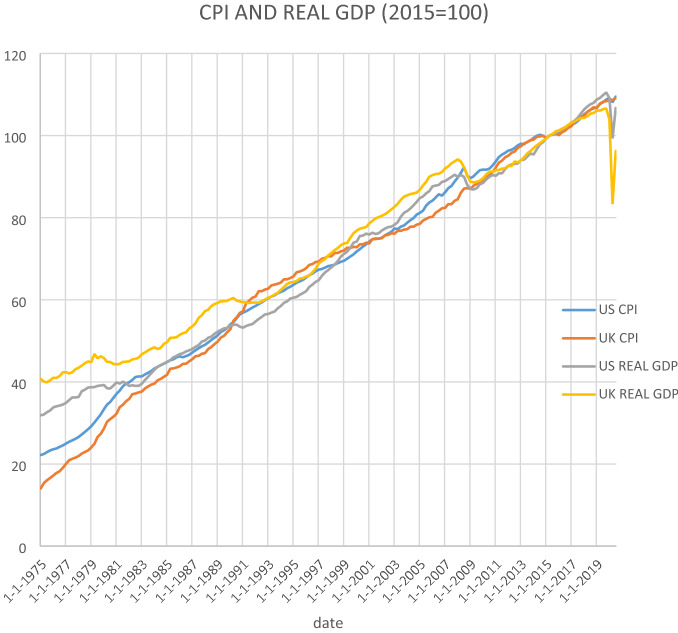
CPI and real GDP (2015 = 100)

The quarterly series used are the consumer price index (CPI) and real gross domestic product (GDP) (index 2015 = 100) for both the UK and the US. The sample period goes from 1975Q1 to 2020Q2. The data sources are the OECD Statistics for the CPI series, Eurostat for UK real GDP and the US Bureau of Economic Analysis for US real GDP. Figure 1 plots both the UK and the US series. They all exhibit very similar behaviour, namely they are upward trending but experience a sharp fall coinciding with the Covid-19 pandemic.

As a first step we carry out univariate analysis, and estimate the following model:

3 $$y_{t} \, = \,\alpha + \,\beta \,t + x_{t} \,,\,(1\,\, - \,\,L)^{d} x_{t} \, = \,u_{t} ,\,\,\,\,t\, = \,1\,,\,\,2\,,\,\,...\,,$$

where *y*_*t*_ is the time series under examination and u_t_ is assumed to be *I* (0) and to follow alternatively a white noise or an autocorrelation process, in the latter case specifically the non-parametric model of Bloomfield (1973).^3^

Table 1 reports the estimated values of d for the raw data and the three standard specifications considered in the unit root literature (Schmidt and Phillips 1992), namely: (i) no deterministic terms, (ii) an intercept, and (iii) and intercept and a linear time trend. The best model is selected on the basis of the statistical significance of the estimated regressors as indicated by their t-statistics. Table 2 shows the parameter estimates for all three specifications. Tables 3 and 4 provide the corresponding information for the logged series.

**Table 1:** Estimates of *d*: raw data

Series	No deterministic terms	An intercept	An intercept and a linear trend
(i) White noise errors			
CPI UK	1.05 (0.94, 1.17)	1.17 (1.09, 1.26)	**1.12 (1.06, 1.20)**
CPI USA	0.98 (0.87, 1.13)	1.21 (1.07, 1.36)	**1.16 (1.04, 1.30)**
REAL GDP UK	0.87 (0.73, 1.08)	0.80 (0.74, 0.87)	**0.74 (0.65, 0.85)**
REAL GDP USA	0.98 (0.85, 1.16)	0.86 (0.82, 0.93)	**0.81 (0.72, 0.92)**
(ii) Autocorrelated errors			
CPI UK	1.04 (0.83, 1.26)	1.51 (1.36, 1.70)	**1.41 (1.25, 1.60)**
CPI USA	0.84 (0.57, 1.11)	1.01 (1.01, 1.29)	**0.97 (0.82, 1.17)**
REAL GDP UK	0.41 (0.36, 0.52)	0.94 (0.85, 1.10)	**0.89 (0.65, 1.11)**
REAL GDP USA	0.60 (0.52, 0.77)	0.97 (0.90, 1.10)	**0.92 (0.76, 1.10)**

In parentheses, the 95% confidence band for the non-rejection values of d at the 95% level. In bold, the selected model for each series on the basis of the statistical significance of the regressors

**Table 2:** Estimated coefficients of the models from Table 1

Series	*d*	Intercept (*t*-statistic)	Time trend (*t*-statistic)
(i) White noise errors			
CPI UK	1.12 (1.06, 1.20)	13.4034 (33.20)	0.5298 (9.93)
CPI USA	1.16 (1.04, 1.30)	21.7330 (58.79)	0.4792 (8.08)
REAL GDP UK	0.74 (0.65, 0.85)	39.9207 (23.73)	0.3385 (8.68)
REAL GDP USA	0.81 (0.72, 0.92)	31.3209 (32.41)	0.4176 (13.97)
(ii) Autocorrelated errors			
CPI UK	1.41 (1.25, 1.60)	13.2100 (34.52)	0.6297 (3.31)
CPI USA	0.97 (0.82, 1.17)	21.7772 (59.53)	0.4650 (2.56)
REAL GDP UK	0.89 (0.65, 1.11)	40.2600 (22.66)	0.3207 (4.09)
REAL GDP USA	0.92 (0.76, 1.10)	31.4018 (31.65)	0.4143 (8.24)

In parentheses in column 2 the 95% confidence band for the non-rejection values of *d*, and in columns 3 and 4 the *t*-statistics

**Table 3:** Estimates of *d*: logged data

Series	No deterministic terms	An intercept	An intercept and a linear trend
(i) White noise errors			
CPI UK	1.01 (0.92, 1.13)	1.45 (1.37, 1.55)	**1.35 (1.29, 1.41)**
CPI USA	0.98 (0.89, 1.10)	1.56 (1.48, 1.67)	**1.46 (1.39, 1.56)**
REAL GDP UK	0.98 (0.88, 1.11)	0.83 (0.77, 0.91)	**0.81 (0.74, 0.90)**
REAL GDP USA	0.98 (0.89, 1.11)	0.97 (0.87, 1.11)	**0.98 (0.91, 1.09)**
(ii) Autocorrelated errors			
CPI UK	1.00 (0.84, 1.18)	1.55 (1.43, 1.70)	**1.40 (1.32, 1.54)**
CPI USA	0.95 (0.80, 1.15)	1.56 (1.43, 1.73)	**1.44 (1.33, 1.60)**
REAL GDP UK	0.89 (0.73, 1.09)	1.01 (0.87, 1.19)	**1.00 (0.83, 1.19)**
REAL GDP USA	0.93 (0.77, 1.13)	1.10 (0.92, 1.31)	**1.05 (0.90, 1.23)**

In parentheses, the 95% confidence band for the non-rejection values of d at the 95% level. In bold, the selected model for each series on the basis of the statistical significance of the regressors

**Table 4:** Estimated coefficients of the models from Table 3

Series	*d*	Intercept (*t*-statistic)	Time trend (*t*-statistic)
(i) White noise errors			
CPI UK	1.35 (1.29, 1.41)	2.6008 (276.26)	0.0197 (5.41)
CPI USA	1.46 (1.39, 1.56)	3.0861 (632.66)	0.0120 (4.13)
REAL GDP UK	0.81 (0.74, 0.90)	3.6998 (184.80)	0.0050 (8.15)
REAL GDP USA	0.98 (0.91, 1.09)	3.4544 (298.65)	0.0066 (8.55)
(ii) Autocorrelated errors			
CPI UK	1.40 (1.32, 1.54)	2.5973 (262.91)	0.0232 (4.93)
CPI USA	1.44 (1.33, 1.60)	3.0862 (613.01)	0.0118 (4.22)
REAL GDP UK	1.00 (0.83, 1.19)	3.7020 (181.57)	0.0047 (3.13)
REAL GDP USA	1.05 (0.90, 1.23)	3.4534 (299.08)	0.0066 (6.12)

In parentheses in column 2, the 95% confidence band for the non-rejection values of *d*, and in columns 3 and 4 the *t*-statistics

Concerning the original series, Table 1 shows that a time trend is required in all cases regardless of the assumption made about the process driving the errors; further, the estimates of d are higher for CPI than for real GDP in both cases. More specifically, the CPI series are characterised by orders of integration above 1 in both countries with white noise errors and in the case of the UK also with autocorrelated errors; however, the unit root null cannot be rejected for the US CPI series with autocorrelated errors. As for real GDP, the estimated value of d is below 1 and the series exhibits mean reversion with white noise errors in both countries; however, under the assumption of autocorrelated errors, the *I* (1) hypothesis cannot be rejected despite the fact that the estimated values of d are still below 1. Table 2 displays the estimated coefficients; as can be seen, there is a positive time trend in all cases.

As for the logged series (see Tables 3 and 4) the orders of integration are significantly higher than 1 in the case of CPI, (with values between 1.35 and 1.46) whilst the unit root null cannot be rejected for real GDP, except for the UK series under the assumption of white noise errors, when mean reversion (*d* < 1) is found.

As explained before, the fact that the price and output series do not have the same order of integration in either country implies that cointegration analysis cannot be carried out. Therefore, we examine their linkages by estimating a model in which one of the two is treated as weakly exogenous as follows:

4 $$y_{1t} = \,\alpha \, + \,\beta \,y_{2t - k} + \,x_{t} \,,(1\,\, - \,\,L)^{d} x_{t} \, = \,u_{t} ,\,\,\,\,\,\,t = 1\,,\,\,2\,,\,\,...\,,$$

for k = 1, 2, and 3, where y_1t_ and y_2t_ stand for log CPI and log real GDP, respectively, in Tables 5 and 6 for both the UK and the US, whilst in Tables 7 and 8 the opposite holds, namely *y*_1*t*_ stands for log real GDP and *y*_2*t*_ for log CPI, respectively, for both countries.

**Table 5:** Estimated coefficients from the regression of log CPI/log RGDP_(UK case)

Lag order	*d* (95% confidence band)	Intercept (*t*-statistic)	Slope coefficient (*t*-statistic)
(i) White noise errors			
*k* = 1	1.46 (1.39, 1.56)	2.5306 (17.65)	0.0491 (1.27)
*k* = 2	1.44 (1.37, 1.52)	3.0628 (9.78)	− 0.0818 (− 0.97)
*k* = 3	1.43 (1.37, 1.52)	2.8246 (8.65)	− 0.0087 (-0.09)
*k* = 4	1.43 (1.36, 1.51)	2.7566 (28.45)	0.0192 (0.21)
(ii) Autocorrelated errors			
*k* = 1	1.73 (1.53, 1.99)	2.4636 (17.23)	0.0667 (1.73)
*k* = 2	1.64 (1.52, 1.82)	2.9761 (9.42)	− 0.0058 (− 0.68)
*k* = 3	1.67 (1.51, 1.85)	2.8722 (9.75)	− 0.0021 (− 0.27)
*k* = 4	1.66 (1.50, 1.90)	2.8347 (9.60)	− 0.0020 ( − 0.02)

In parentheses in column 2, the 95% confidence band for the non-rejection values of *d*, and in columns 3 and 4 the *t*-statistics

**Table 6:** Estimated coefficients from the regression of log CPI/log RGDP_(US case)

Lag order	*d* (95% confidence band)	Intercept (*t*-statistic)	Slope coefficient (*t*-statistic)
(i) White noise errors			
*k* = 1	1.56 (1.48, 1.64)	3.1799 (22.58)	− 0.0336 (-0.55)
*k* = 2	1.57 (1.38, 1.66)	3.3794 (17.68)	− 0.0743 (-1.35)
*k* = 3	1.56 (1.48, 1.66)	3.09336 (16.04)	0.0144 (0.26)
*k* = 4	1.56 (1.48, 1.66)	2.7673 (14.48)	**0.1122 (2.03)**
(ii) Autocorrelated errors			
*k* = 1	1.53 (1.42, 1.70)	2.9622 (21.03)	0.0406 (0.99)
*k* = 2	1.57 (1.45, 1.74)	3.3794 (17.91)	− 0.0743 (-1.36)
*k* = 3	1.59 (1.43, 1.78)	3.1079 (16.28)	0.0102 (0.18)
*k* = 4	1.57 (1.41, 1.77)	2.7718 (14.59)	**0.1109 (2.02)**

In parentheses in column 2, the 95% confidence band for the non-rejection values of *d*, and in columns 3 and 4 the *t*-statistics. In bold, the significant slope coefficients at the 5% level

**Table 7:** Estimated coefficients from the regression of log RGDP/log CPI_(UK case)

Lag order	*d* (95% confidence band)	Intercept (*t*-statistic)	Time trend (*t*-statistic)
(i) White noise errors			
*k* = 1	1.03 (0.94, 1.17)	3.3462 (13.38)	0.1301 (1.37)
*k* = 2	1.02 (0.93, 1.16)	3.3309 (13.60)	0.1345 (1.45)
*k* = 3	1.02 (0.93, 1.15)	3.3397 (13.60)	0.1355 (1.46)
*k* = 4	1.02 (0.94, 1.16)	3.4060 (13.89)	0.1164 (1.25)
(ii) Autocorrelated errors			
*k* = 1	1.03 (0.94, 1.16)	3.3249 (13.62)	0.1382 (1.50)
*k* = 2	1.02 (0.92, 1.15)	3.3309 (13.62)	0.1345 (1.45)
*k* = 3	1.02 (0.92, 1.16)	3.3397 (13.60)	0.1355 (1.46)
*k* = 4	1.02 (0.94, 1.16)	3.4060 (13.89)	0.1165 (1.25)

In parentheses in column 2, the 95% confidence band for the non-rejection values of *d*, and in columns 3 and 4 the *t*-statistics. In bold, the significant slope coefficients at the 5% level

**Table 8:** Estimated coefficients from the regression of log RGDP/log CPI_(US case)

Lag order	*d* (95% confidence band)	Intercept (*t*-statistic)	Time trend (*t*-statistic)
(i) White noise errors			
*k* = 1	1.08 (0.98, 1.36)	2.7601 (9.10)	**0.2275 (2.32)**
*k* = 2	1.08 (0.98, 1.35)	2.6888 (8.81)	**0.2561 (2.60)**
*k* = 3	1.08 (0.99, 1.35)	2.6097 (8.53)	**0.2859 (2.89)**
*k* = 4	1.09 (0.98, 1.35)	2.9327 (9.38)	**0.1890 (1.87)**
(ii) Autocorrelated errors			
*k* = 1	1.08 (0.97, 1.37)	2.7601 (9.11)	**0.2275 (2.32)**
*k* = 2	1.08 (0.98, 1.38)	2.6888 (8.81)	**0.2561 (2.60)**
*k* = 3	1.08 (0.98, 1.37)	2.6097 (8.53)	**0.2859 (2.89)**
*k* = 4	1.09 (0.97, 1.38)	2.9327 (9.38)	**0.1890 (1.87)**

In parentheses in column 2, the 95% confidence band for the non-rejection values of *d*, and in columns 3 and 4 the *t*-statistics. In bold, the significant slope coefficients at the 5% level

Concerning the regression of log CPI on log real GDP, in the UK case (Table 5) the estimated values of *d* are much higher than 1 regardless of the lag length and range between 1.43 (*k* = 3 and 4 with white noise errors) and 1.73 (*k* = 1 with Bloomfield errors); however, the slope coefficient is not significantly different from zero in any single case. As for the US results (Table 6), the estimates of d are again much higher than 1 (between 1.53 and 1.59), but the slope coefficient is now significant for *k* = 4, which might reflect a seasonal effect given the quarterly frequency of the series examined.

When regressing instead log real GDP against log CPI, in the UK case (Table 7) the estimates of d are slightly above 1, the unit root null hypothesis cannot be rejected in any single case, and the slope coefficient is not significant in any case. By contrast, in the US case (Table 8) there is a positive relationship between previous values of CPI and real GDP, which suggests a lagged impact of demand shocks.

## Conclusions

This paper applies a fractional integration approach to UK and US quarterly data on prices and output from 1975Q1 to 2020Q2 to analyse the stochastic behaviour of these two variables and their long-run relationship in both economies—unlike most of the existing literature that focuses instead on their correlation over the business cycle. The univariate analysis indicates that all series are highly trended and persistent, exhibiting high degrees of integration, especially in the case of CPI.

As for their linkages, since the two variables have different degrees of integration in each of the two countries, fractional cointegration tests cannot be carried out. We assume instead weak exogeneity of each of them in turn and examine causality by testing for the significance of the lagged values of the variable treated as exogenous. We find that the only significant relationship implies the existence of a lagged effect of prices on output in the case of the US, which suggests a dominant role for demand shocks. An alternative approach could be based on the AutoRegressive Distributed Lag (ARDL) model (see Pesaran and Shin 1999), which does not require the assumption of equal orders of integration. However, this framework has yet to be extended to the case of fractional integration. Work in this direction is currently in progress. In addition, it is noteworthy that if possible nonlinearities and/or structural breaks are not taken into account they may produce spurious long-memory relations (see, e.g., Diebold and Inoue, 2001; Granger and Hyung 2004; etc. For this reason break tests in *I* (*d*) contexts have recently been developed (see, e.g., Gil-Alana 2008; Mensi et al. 2014; etc.) these could be used in future work to investigate such issues further.

## Data Availability

Data will be available from the authors upon reasonable request.

## Footnotes

1 The most commonly used are the ADF test (Dickey and Fuller 1979) and other tests developed by Phillips and Perron (1988), Kwiatkowski et al. (1992), Elliot et al. (Elliot et al. 1996), Ng and Perron (2001), etc.

2 See Diebold and Rudebusch (1991), Hassler and Wolters (1994), Lee and Schmidt (1996), etc.

3 3This is a non-parametric way of describing the *I* (0) error term that produces autocorrelations decaying at an exponential rate as in the AR case.

## Code availability

Standard codes available on the Internet have been used for the analysis.
